# Genome-wide hairpins datasets of animals and plants for novel miRNA prediction

**DOI:** 10.1016/j.dib.2019.104209

**Published:** 2019-07-03

**Authors:** L.A. Bugnon, C. Yones, J. Raad, D.H. Milone, G. Stegmayer

**Affiliations:** Research Institute for Signals, Systems and Computational Intelligence sinc(i) (FICH-UNL/CONICET), Ciudad Universitaria, Santa Fe, Argentina

**Keywords:** Bioinformatics, miRNA prediction, Genome-wide data, miRNA features

## Abstract

This article makes available several genome-wide datasets, which can be used for training microRNA (miRNA) classifiers. The hairpin sequences available are from the genomes of: *Homo sapiens, Arabidopsis thaliana, Anopheles gambiae, Caenorhabditis elegans* and *Drosophila melanogaster*. Each dataset provides the genome data divided into sequences and a set of computed features for predictions. Each sequence has one label: i) “positive”: meaning that it is a well-known pre-miRNA, according to miRBase v21; or ii) “unlabeled”: indicating that the sequence has not (yet) a known function and could be a possible candidate to novel pre-miRNA. Due to the fact that selecting an informative feature set is very important for a good pre-miRNA classifier, a representative feature set with large discriminative power has been calculated and it is provided, as well, for each genome. This feature set contains typical information about sequence, topology and structure. Dataset was publically shared in https://sourceforge.net/projects/sourcesinc/files/mirdata/.

**Table:** Specifications table

*Subject area*	*Bioinformatics*
*More specific subject area*	*Pre-miRNA prediction*
*Type of data*	*Tabular data and genomic sequences*
*How data was acquired*	*Own genome-wide hairpins sequence extractor; and feature extractor miRNAfe*[3]
*Data format*	*Features in comma-separated-value files and genomic sequences in FASTA format.*
*Data source location*	*Argentina.*
*Data accessibility*	*Public repository:*https://sourceforge.net/projects/sourcesinc/files/mirdata/

**Table:** 

**Value of the data** • A real-life benchmark dataset for training pre-miRNA classifiers, of several and complete genomes, is provided. • Sequences of each genome is fully labeled and feature extraction of the sequences, which is a very time-consuming task, has been done and is provided as well. • For the first time, several animals and plants sequences, extracted from a complete genome and not just portions of it, are fully available to the academic community. • These datasets can be used for a fair and accurate comparison of pre-miRNA classifiers on real data, in order to guarantee reproducible results. • Unlike other existing public datasets, this new one can be used in a realistic and complete genome-wide prediction task, avoiding the manual definition of artificial negative samples for training classifiers. • To the best of our knowledge, this is the first time that such a variety of hairpin sequences as well as corresponding feature extracted data, are made freely available to the research community.

## 1 Data

In this work we provide genome-wide hairpins datasets of animals and plants, which can be used as benchmark data for training and testing pre-miRNA predictors. Data consists of a set of FASTA files with folded hairpins sequences of 5 complete genomes:^1^

• *Homo sapiens (hsa),*
• *Arabidopsis thaliana (ath),*
• *Anopheles gambiae (aga),*
• *Caenorhabditis elegans (cel)*, and
• *Drosophila melanogaster* (*dme*).

**Fig. 1:**
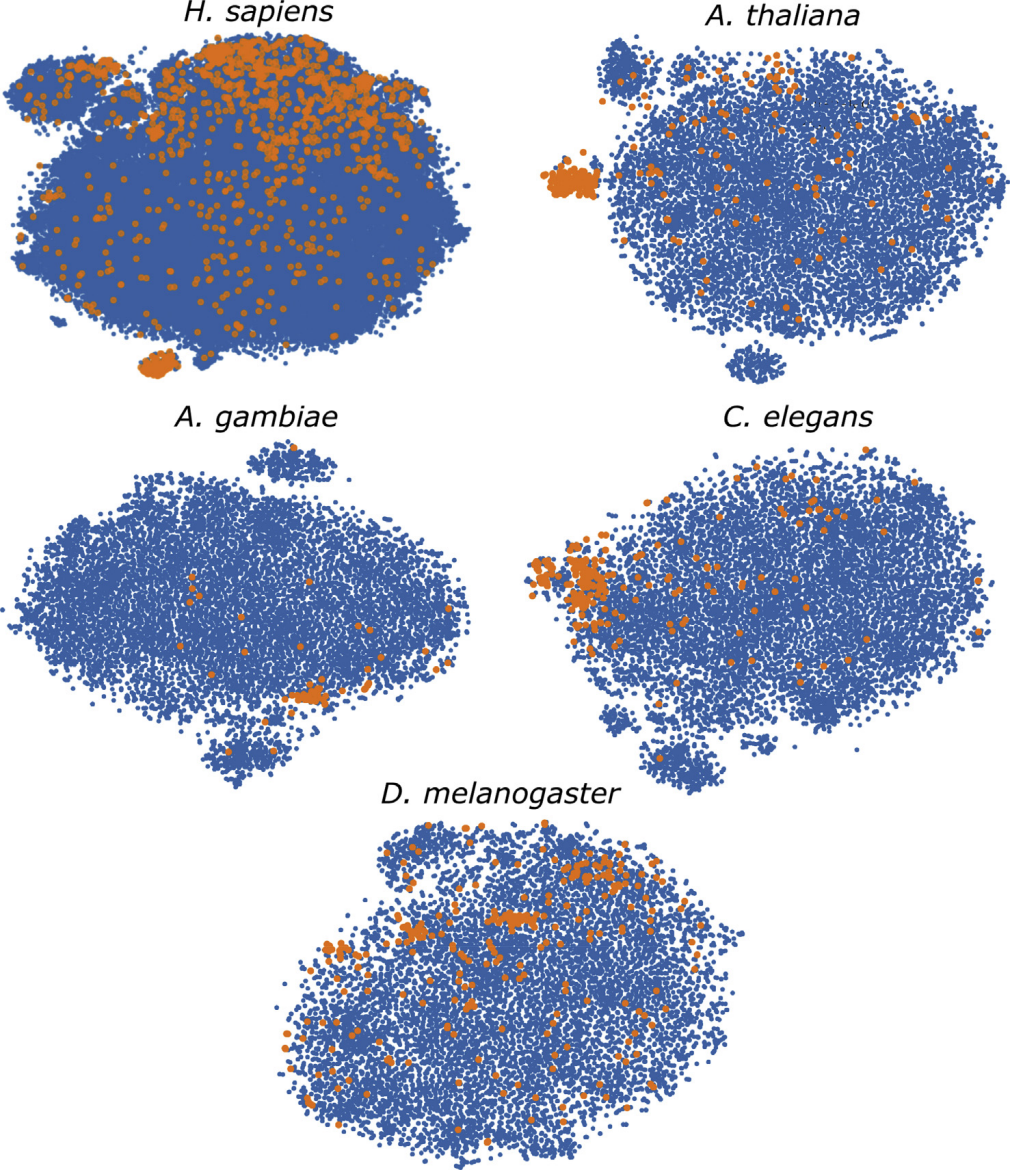
t-SNE projection with the well-known pre-miRNAs (in orange) and a random set of unlabeled sequences (in blue) for each genome. Sequences that are closer in the projected feature space have more similar features.

**Fig. 2:**
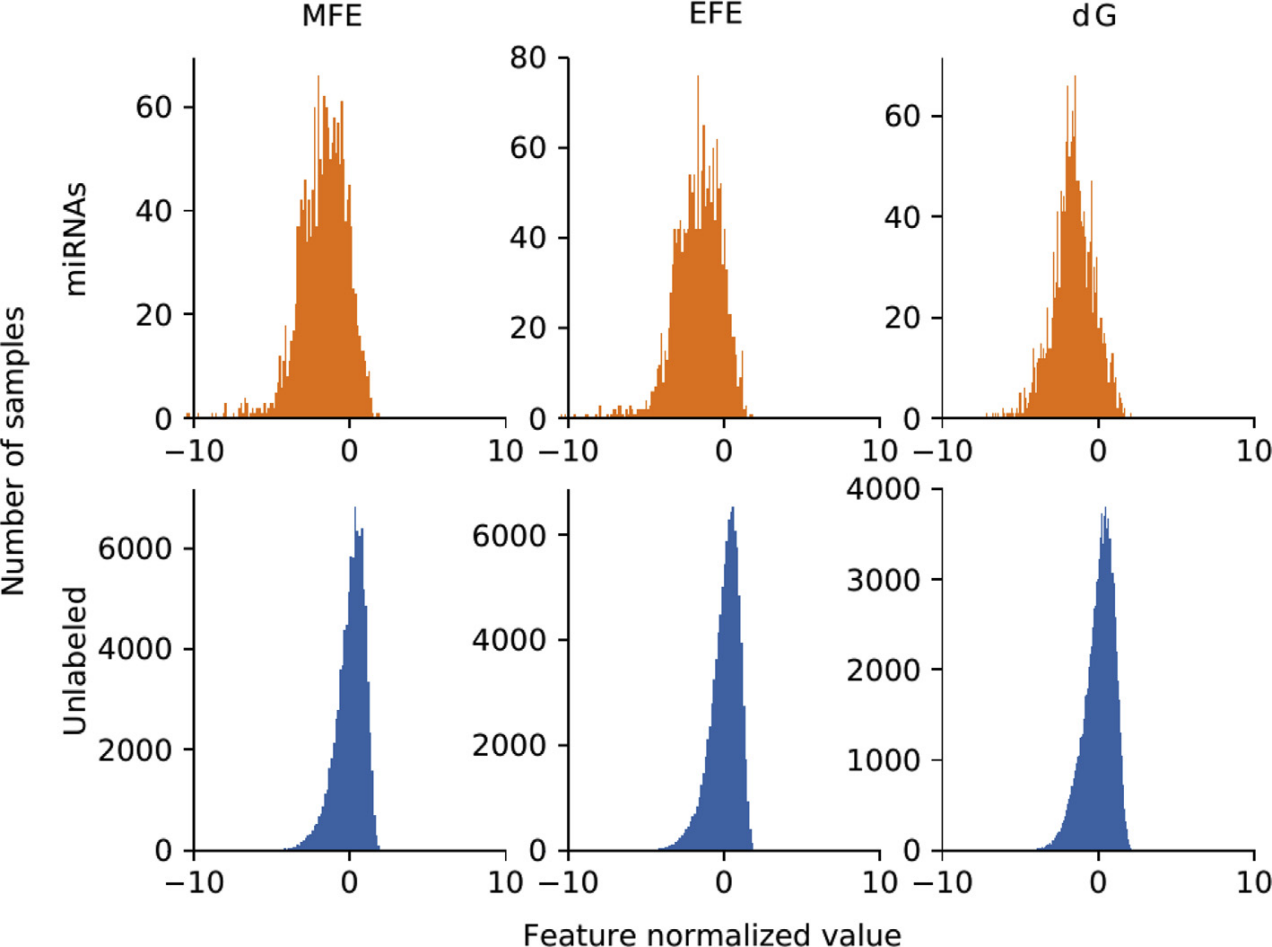
Histograms of the top 3 features distributions in *H. sapiens*: minimum free energy, normalized ensemble free energy (RNAfold) and minimum free energy normalized by length. Well known miRNAs, in orange, have a slightly different distribution but highly overlapped when compared with other sequences (in blue).

**Fig. 3:**
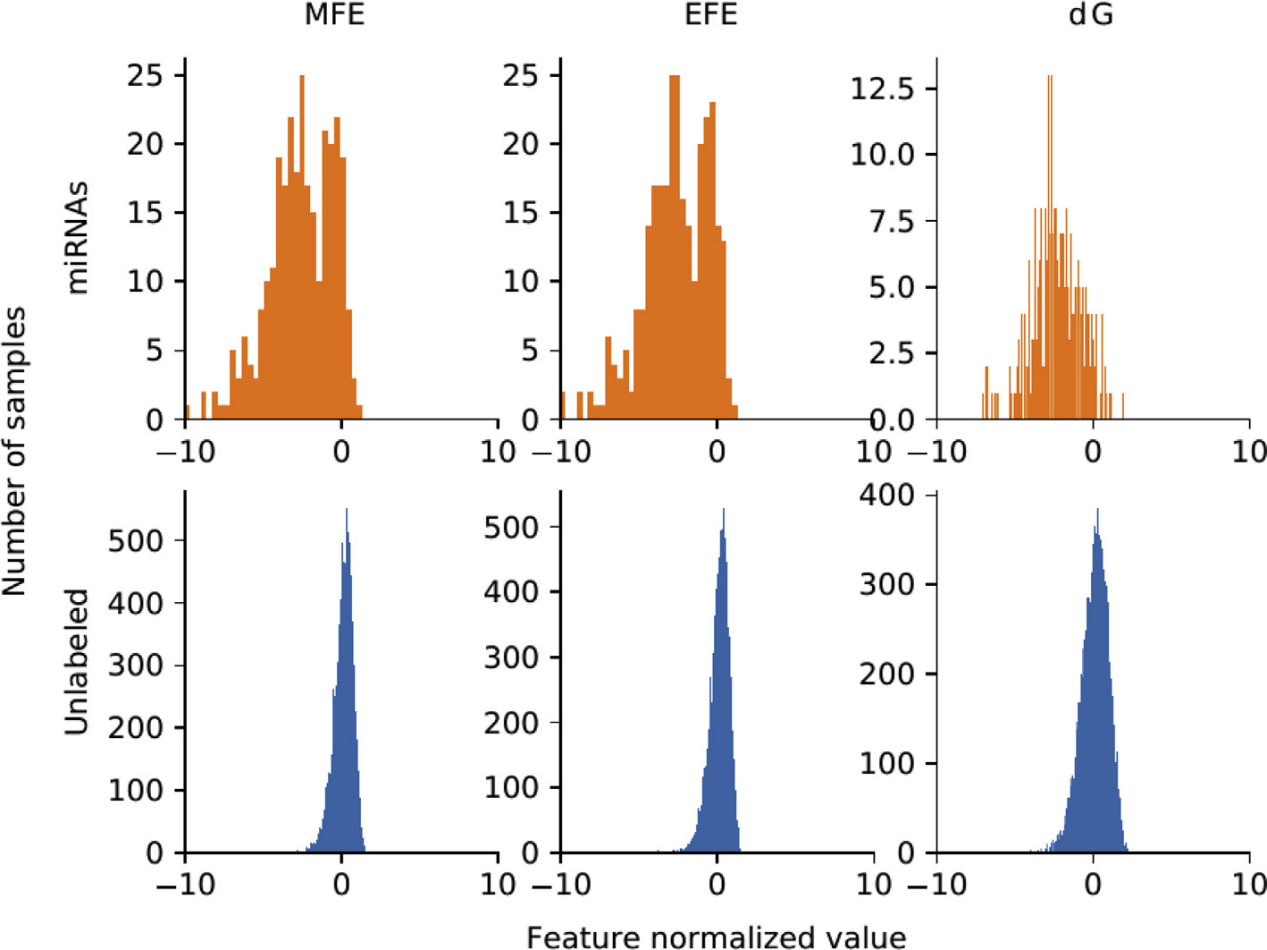
Histograms of the top 3 features distributions in *A. thaliana*.

**Fig. 4:**
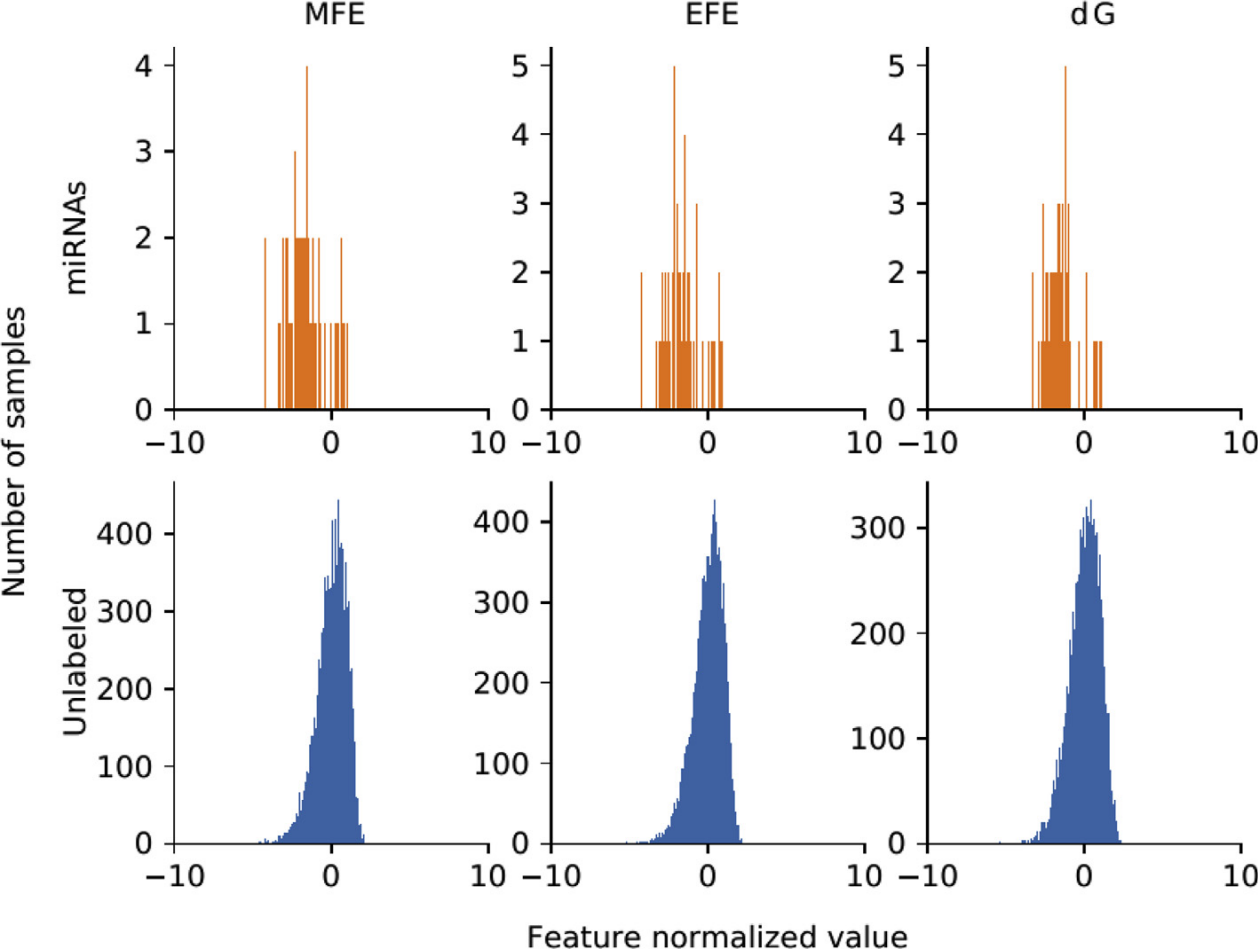
Histograms of the top 3 features distributions in *A. gambiae*.

**Fig. 5:**
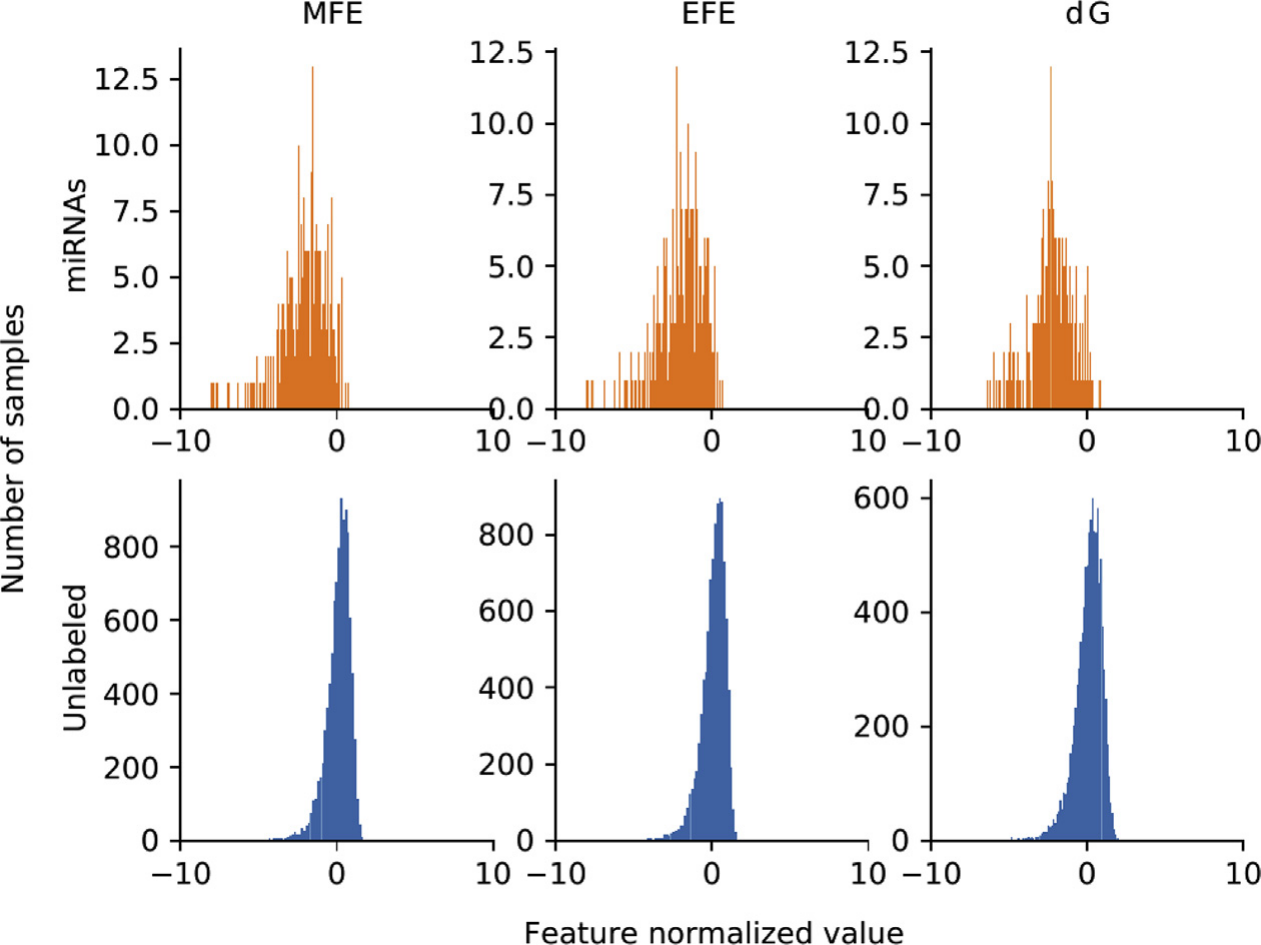
Histograms of the top 3 features distributions in *C. elegans*.

**Fig. 6:**
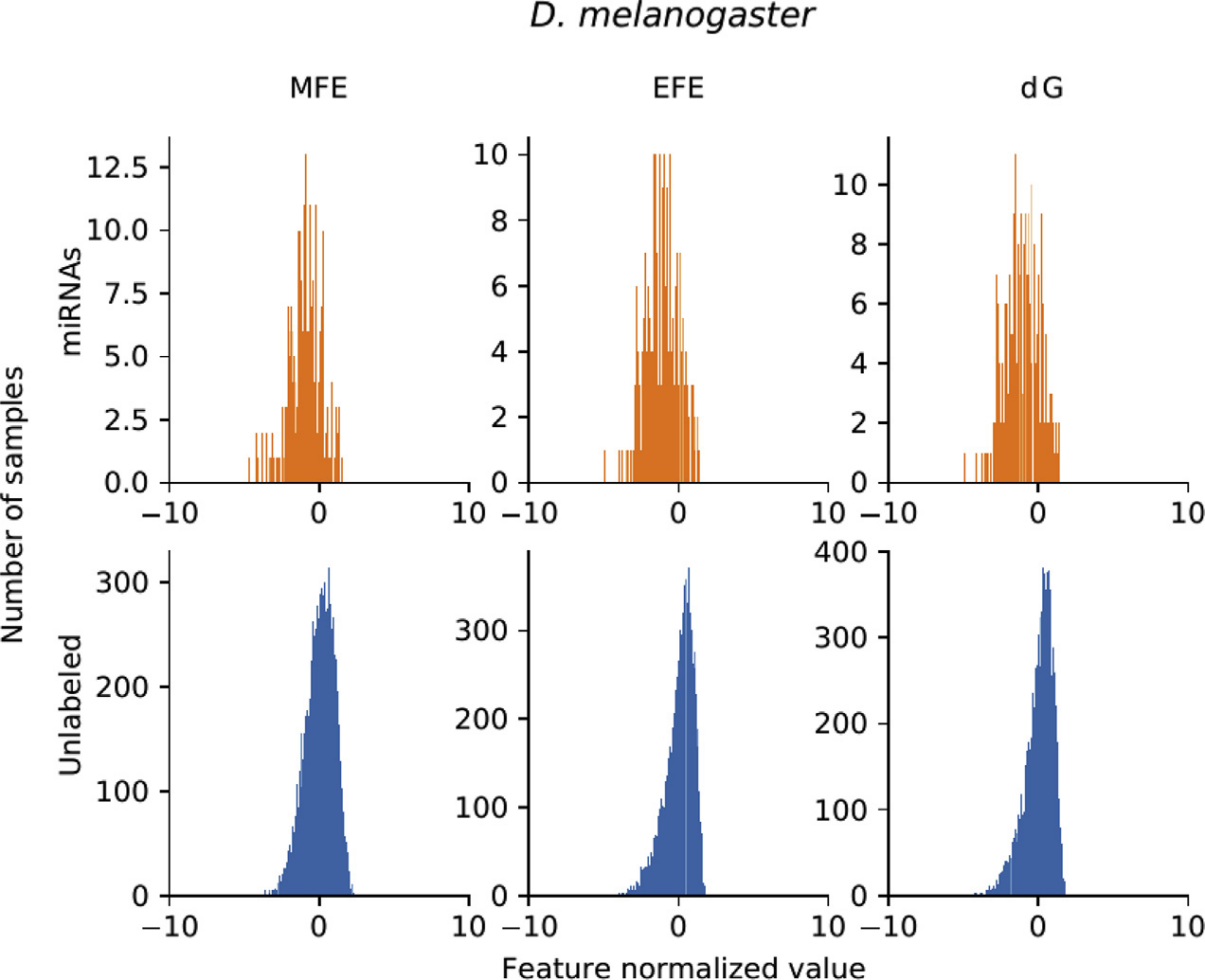
Histograms of the top 3 features distributions in *D. melanogaster*.

For each genome, there is a set of well known miRNAs sequences, and a larger set of unknown sequences that fold into hairpin structures. Table 1 shows the details of the sequences that have been extracted. For each genome (first column) in the rows, the second column indicates the total number of sequences extracted, which can form hairpins; the third column shows the number of known miRNAs found for each corresponding species. A large number of discriminative features were computed (77 dimensions in total) and stored in.csv files for each genome. The features are listed in Table 2: each row has the feature name, description and dimension (the number of values computed for each feature). A representation of the distribution of the features among positive and unlabeled examples is depicted in Fig. 1. The features values were normalized subtracting the mean and dividing by the corresponding variance and then a t-Distributed Stochastic Neighbor Embedding (t-SNE) [24] was computed. This method generates a 2D projection of the sequences considering the samples neighborhood, based on the similarity of their features. Moreover, Fig. 2, Fig. 3, Fig. 4, Fig. 5, Fig. 6 show the histograms of the normalized features.

**Table 1:** Number of stem loops and pre-miRNAs in each genome.

Species	Extracted hairpins	miRNAs
*H. sapiens*	48,181,565	1710
*A. thaliana*	1,355,663	304
*A. gambiae*	4,268,407	66
*C. elegans*	1,737,349	249
*D. melanogaster*	2,066,807	307

**Table 2:** Features calculated for each sequence.

Feature name	Description	Dimension
nt_proportion	Ratio of each base in the sequence (A, C, G and T)	4
dinucleotide_proportion	Ratio of dinucleotide elements of each kind, making 16 Features for the possible binary combinations of the 4 nucleotides	16
gc_content	Proportion of guanine and cytosine on the sequence	1
gc_ratio	Ratio between guanine and cytosine	1
sequence_length	The length of the sequence	1
stem_number	The number of stem-loops	1
avg_bp_stem	Average of nucleotides per stem	1
longest_stem_length	Longest region where the pairing is perfect	1
terminal_loop_length	Number of nucleotides in the stem region	1
bp_number	Number of base-pairs	1
dP	Number of base pair divided by the nucleotide number	1
bp_proportion	Number of each possible base pair normalized by sequence length	3
bp_proportion_stem	Proportion of base pairs on stems	3
triplets	Frequencies of secondary structure triplets, this is the 32 possible combinations of the 4 nucleotides in a sequence of 3	32
MFE	Minimum free energy	1
EFE	Normalized Ensemble Free Energy calculated with RNAfold (-p option)	1
ensemble_frequency	The frequency of the minimum free energy in the ensemble	1
diversity	Structural diversity calculated with RNAfold (-p option)	1
mfe_efe_difference	Calculated as |MFE-EFE|/*l*	1
dQ	Calculated as 1/*L*$\sum_{i<j}p_{ij}$ log_2_*p*_*ij*_, where *L* is length and *p*_*ij*_ is the probability of pairing of nucleotides *i* and *j*	1
dG	Minimum free energy divided by sequence length	1
MFEI_1_	Ratio between the minimum free energy and the %C+G	1
MFEI_2_	*dG/N*_*s*_, where *N*_*s*_ is the number of stems.	1
MFEI_4_	*MFE/N*_*b*_, where *N*_*b*_ is the total number of base pairs in the secondary structure	1

## 2 Experimental design, materials and methods

The importance of microRNAs (miRNAs) has been largely recognized by the scientific community. MiRNAs on average are about 21 nucleotides long, and take part in the post-transcriptional regulation of gene expression. These short segments of RNA play a role in many fundamental biological processes, such as promoting or inhibiting certain diseases and infections [1]. Precursors of miRNAs (pre-miRNAs, also known as hairpins) are generated during biogenesis and have a very well-known secondary structure: a typical stem-loop structure with few internal loops or asymmetric bulges. Unfortunately, a large amount of hairpin-like structures can be found in a genome [2].

The computational prediction of novel pre-miRNAs involves training a machine learning classifier for identifying candidate sequences for being novel miRNAs. However, to the best of our knowledge, there are no such datasets available. Actually, in most published works, the datasets used for training and testing the prediction methods are manually built, use diverse methodologies according to each study [4], [5], [6], [7], [8], [9], [10], [11], [12], [13], [14], [15], [16], and require a (not negligible) long time. Secondly, it is very hard to fairly compare among different classifiers. Therefore, this makes that published experiments of most pre-miRNA prediction methods cannot be accurately reproduced nor be fully trusted, because the users of those tools cannot obtain the same prediction rates as those published.

In this dataset, we included sequences of model genomes in animals and plants. Although miRNAs may have had a common origin, they had evolved in different ways in the plant and animal kingdoms. The proteins involved in the maturation process of the precursors and the places where it takes place, can be very different. In animals, the transcription of the primary miRNAs (pri-miRNAs) is carried out by RNApol II and RNApol III [17], [18]. After transcription, the pri-miRNAs form stem-loop structures, also called hairpins. These structures are recognized in the nucleus by Drosha and a miRNA precursor (pre-miRNA) is obtained by cleavage. After that, the precursor is exported to the cytoplasm, where it is cut near the terminal loop by the Dicer enzyme, forming a small double-stranded RNA [18]. Some species possess multiple Dicer homologues with different roles. For instance, in *D. melanogaster* and *A. gambiae*, Dicer-1 is required for miRNA biogenesis [19]. Following Dicer processing, miRNA is preferentially loaded onto particular types of AGO proteins and the complementary miRNA sequence is discarded. In *C. elegans*, for example, miRNA duplexes and siRNA duplexes are sorted into ALG-1 and ALG-2 proteins. In humans, by contrast, the four AGO proteins are associated with almost indistinguishable sets of miRNAs because no strict small-RNA-sorting system exists. In plants, the primary miRNAs are transcribed only by RNApol II. In addition, the length of the pri-miRNAs may have a high variation [20]. Unlike the process of the pri-miRNAs in animals, in plants the process of maturation of the miRNAs is carried out completely in the nucleus. This maturation process is not performed by Drosha, because it is not found in plants. Instead, Dicer1-like processes most pri-miRNAs by sequential cleavage in the basal and the apical junctions of the terminal loop [21]. Following this processing, the duplex miRNAs is exported to the cytoplasm. In the cytoplasm, miRNAs are loaded onto cytoplasmic AGO protein [20]. Thus, since the pre-miRNAs biogenesis is different in the animal and plant kingdom, we have included sequences of the species considered as model genomes in these kingdoms.

## 3 Materials and methods

Each complete raw genome was downloaded from ftp://ftp.ncbi.nlm.nih.gov/genomes/. The input genome-wide data (a multi-fasta file named, for example, genome.fa) is pre-processed with our open-source toolkit HextractoR,^2^ which automatically extracts and folds all hairpin sequences from raw genome-wide data. It predicts the secondary structure of several overlapped segments, with longer length than the mean length of sequences of interest for the species under processing, ensuring that no one is lost nor inappropriately cut. Then, the prediction of the secondary structures of the sequences obtained was done with the minimum free energy algorithm [22] of RNAfold. After that, miRNAfe [3] was used to extract features for each sequence. Finally, BLAST matching between the extracted sequences and the known miRNAs in miRBase [23] has been done, in order to automatically identify and label those sequences that are, actually, well-known pre-miRNAs.

Each genome has been cut into overlapping windows of a large length (500 nt). This window has been chosen in order to correctly capture a complete hairpin, but also to take into account the neighborhood of any possible hairpin when estimating the secondary structure. This is very important since the results of estimating a secondary structure can be very much affected by the neighborhood of the sequences. Then, the prediction of the secondary structures of the sequences obtained in the previous windowing step has been done. To do this, the minimum free energy algorithm [5] of RNAfold has been used. This algorithm uses dynamic programming for finding the secondary structure that minimizes the energy released. Those hairpins that did not exceed a minimum length of 60 and level pairing of 16 were eliminated.

In order to obtain sequences with lengths similar to those of the well-known pre-miRNA of the particular genome under analysis (found with BLAST matching of the extracted sequences against miRBase), the extracted sequences were trimmed trying to optimize the normalized Minimum Free Energy (NMFE) by the sequence length. The following rules have been applied to achieve this:

1. Each sequence extracted not having a specified minimum length, according to the miRNAs of the genome under analysis, was discarded. This was done in order to ensure that the secondary structure had sufficient length to be a pre-miRNA.
2. The cuts were made in the first unpaired nucleotide of an internal loop or bulge of the secondary structure (starting from the main loop) that passes the minimum length specified. That is, from all unpaired nucleotides, only the ones that are at a certain distance from the main loop are candidates to be a cutting point. It is likely that cutting the sequence at those points will result in a structure with lower NMFE. Moreover, the smaller the length of the sequence (independently of the pairing), the higher the NMFE. Therefore, a loop/bulge closer to the main loop is preferred.

Repeated sequences were eliminated to avoid extra computational cost and because they might also disturb the results of the prediction algorithms, since each repeated sequence increases its relevance for the predictor. Repetitions may appear due to the overlapping in windowing. These repeated sequences appear consecutively and they can be almost identical. To eliminate them, a comparison between each sequence and the last extracted sequence is made. If one of the sequences contains the other one, the shortest one is discarded. Finally, for labeling the sequences obtained, BLAST matching is done against miRBase. The sequences that match, are labeled as positive class (pre-miRNAs).

A characterization of the features of each dataset has been done. A t-SNE projection is shown in Fig. 1. The well-known pre-miRNAs sequences are highlighted in orange, and plotted together with the unlabeled samples in blue. It can be seen that there are some known miRNAs that are close in the projected space. However, there are also many positive samples scattered all over the feature space, showing that accurate prediction is, indeed, a challenging task. This is especially notorious in the *H. sapiens* and *D. melanogaster* genomes, which have a very large number of sequences and several well-known miRNAs.

A further insight of the relevance of each feature was done ranking the features according to its importance for classification. Training a random forest [7] with 10 trees, it is possible to see which features are the best ones to separate positive versus unlabeled samples. Taking the average rank across all the genomes, the top-5 most informative features are shown in Table 3. It can be seen that the normalized ensemble free energy (EFE), the minimum free energy (MFE) and its value normalized by length (dG) are the most important features, since those features reflect the stability of the hairpin secondary structure.

**Table 3:** Feature relevance.

Feature	Average rank
MFE	0.40
EFE	4.20
dG	6.60
triplets_0_	9.60
MFEI_4_	9.80

Fig. 2, Fig. 3, Fig. 4, Fig. 5, Fig. 6 show the histograms of the normalized features, but now analyzed with the top-3 most interesting features of Table 3. They show that features distribution is, indeed, different among positive and unlabeled classes. However, there is a significant overlapping among them, which makes the prediction a challenging task for simple classifiers. This is one of the main motivation for making available to the research community these benchmark datasets: helping and giving support to the proposal of novel and more advanced prediction methods, which could be now fairly compared on the same experimental conditions, such as in [16].

## Footnotes

2 https://cran.r-project.org/web/packages/HextractoR/index.html.
